# Molecular Modelling a Key Method for Potential Therapeutic Drug Discovery

**DOI:** 10.26717/BJSTR.2021.37.006000

**Published:** 2021-07-23

**Authors:** Omar Karkoutly, Anupam Dhasmana, Vijian Dhevan, Subhash C. Chauhan, Manish K. Tripathi

**Affiliations:** 1 Department of Biology, College of Sciences, The University of Texas Rio Grande Valley, McAllen, TX 78539, USA; 2 South Texas Center of Excellence in Cancer Research, School of Medicine, University of Texas Rio Grande Valley, McAllen TX 78504, USA; 3 Valley Baptist Hospital, Harlingen, USA; 4 Department of Surgery, School of Medicine, University of Texas Rio Grande Valley, Edinburg, TX 78501, USA; 5 Department of Immunology and Microbiology, School of Medicine, University of Texas Rio Grande Valley, USA

**Keywords:** Protein Drug interaction, Drug discovery, High-Throughput Virtual Screenings

## Abstract

The well-defined and characterized 3D crystal structure of a protein is important to explore the topological and physiological features of the protein. The distinguished topography of a protein helps medical chemists design drugs on the basis of the pharmacophoric features of the protein. Structure-based drug discovery, specifically for pathological proteins that cause a higher risk of disease, takes advantage of this fact. Current tools for studying drug-protein interactions include physical, chromatographic, and electrophoretic methods. These techniques can be separated into either non-spectroscopic (equilibrium dialysis, ultrafiltration, ultracentrifugation, etc.) or spectroscopic (Fluorescence spectroscopy, NMR, X-ray diffraction, etc.) methods. These methods, however, can be time-consuming and expensive. On the other hand, *in silico* methods of analyzing protein-drug interactions, such as docking, molecular simulations, and High-Throughput Virtual Screenings (HTVS), are heavily underutilized by core drug discovery laboratories. These kinds of approaches have a great potential for the mass screening of potential small drugs molecules. Studying protein-drug interactions is of particular importance for understanding how the structural conformation of protein elements affect overall ligand binding affinity. By taking a bioinformatics approach to analyzing drug-protein interactions, the speed with which we identify potential drugs for genetic targets can be greatly increased.

## Introduction

Cancer is the second leading cause of death in the United States and serves as a great barrier to increasing life expectancy in many countries around the world [1]. In fact, below the age of 70, cancer is the first or second leading cause of death in 112 of 183 countries and is the third or fourth cause of death in another 23 countries [2]. Although the overall incidence and cancer mortality rate has been greatly reduced over the past couple of years due to advancements in early detection screenings and treatment options, cancer remains prevalent [3]. Factors that increase risk of cancer, like obesity, diabetes, and aging, are on the rise, and as a result, people are at a higher risk of getting cancer, especially in the United States [4]. Furthermore, treatment options are becoming more limited due to the robust characteristics of aggressive cancer types that allows them to obtain drug resistance. This is especially true for the adolescence and young adult (AYA) population, as from 2006–2017, an increase in overall cancer incidence was seen, which is bad because treating younger cancer patients means that the cancer has more time to build up a resistance to the anticancer drug being used. Because of this, research into the development of new drugs for cancer treatment has been an ongoing effort.

Drug resistance for typical chemotherapeutic treatments is one of the main reasons these kinds of cancer therapies result in failure [5]. Despite the considerable progress being made in targeted cancer therapies, there is no treatment that is 100% effective in eliminating cancers, because of their innate resistance (to a broad range of anticancer drugs) or their acquired resistance (as existing therapies become more effective against them) [6]. One reason for this resistance may be due to cancer cell plasticity, which allows for cancer cells to switch between differentiated (limited tumorigenic potential) and undifferentiated (cancer stem cells) states [7]. Plasticity greatly contributes to tumor heterogeneity, which describes the differences between subpopulations of the same tumor type in different patients and is the reason why differential responses to therapies occur [8]. Furthermore, cancers are extremely complex, and their robustness [9,10] allows them to survive, adapt, and maintain their proliferative potential and functionality in the face of any internal or external perturbations (such as against a wide variety of anticancer therapies) [11]. One major solution to this is to develop novel drugs that are either better than their predecessors or that can result in deeper responses from being used sequentially or in combination with existing drugs [12].

## Proteins as Therapeutic Targets

The diagnostic detection and measurement of cancer progression is essential for effective disease management, especially since the early stages of cancer have the highest therapeutic potential [13]. These early stages, however, are typically asymptomatic, and as a result, identifying novel biomarkers of various cancers is essential for early detection [14]. Cancer biomarkers can be any sort of tumor characteristic (like tumor tissue) or bodily response to cancer (like bodily fluids), that help indicate current or future cancer behavior, such as cancer risk, cancer type, and drug or treatment efficacy [15]. Not only can these biomarkers be used in diagnosis and early malignancy detection, but they may also be used as specific drug targets when designing novel anticancer drugs.

A prominent example of cancer biomarkers include the estrogen receptor (ER), the progesterone receptor (PR), and the human epidermal growth factor receptor (HER2), all of which are essential for the standard care of newly diagnosed, recurring, and malignant breast cancer patients [16]. Targeted HER2 drugs, such as Tratsuzumab, a humanized monoclonal antibody against HER2, have been developed and shown to increase time to progression and survival in both early stage and metastatic breast cancers [17]. As cancers continue to develop drug resistance, it becomes more critical to identify new biomarkers and develop more efficient drugs to help combat disease progression [18]. Current tools for doing this are time-consuming and inefficient, but the latest structural modeling tools can make this process easier and faster and can even take advantage of existing drug databases to potentially repurpose known drugs.

## Methods for Drug Identification

The cancer proteome and metabolome (the entire set of proteins or small molecule metabolites, respectively, that are produced by a cancer), can contain important information relating to the discovery of novel biomarkers. Various methods such as electrophoresis, mass-spectroscopy techniques, and protein microarrays can be used to discover novel biomarkers. Additionally, many target-specific immunoassays and immunosensor techniques, including electrochemical, mass-sensitive, and optical have been used for tumor-related biomarker detection [19]. Traditional chemotherapies directly target the DNA of cells, but this can damage healthy cells, so modern approaches to anticancer drugs focus on molecular targeted therapy (i.e. monoclonal antibodies and small molecule inhibitors) to reverse abnormalities in the expression of kinases, tubulin proteins, extracellular matrix components, vascular targets, cancer stem cell pathways, or the tumor microenvironment (like acidity) as possible drug targets so that cancer cells can be selectively killed with a decreased toxicity towards normal cells [20].

Physical methods for studying drug-protein binding have been traditionally divided into either non-spectroscopic (like calorimetry, dialysis, filtration, electrophoresis and centrifugation) or spectroscopic (like UV and visible light absorption, NMR, X-rays, and fluorescence) [21]. These, however, have been replaced with more advanced and efficient methods such as a variety of mass-spectroscopy (MS) techniques including a direct approach, a structural approach, an enzymatic approach, an affinity-based approach, and a global proteomics approach [22]. These various MS approach make it possible to characterize drug target structures, screen large numbers of potential drug candidates (in metabolism and in pharmacokinetic studies), detect drug-target complexes, examine how protein structure is affected by the drug, and monitor the enzymatic activity of the target protein in relation to the drug [23]. Despite these major improvements in analyzing protein-drug interactions, these methods remain complex, time-consuming, and costly [24]. As a result, more convenient tools, such as computational methods and structural modeling, should be used for estimating protein-drug binding affinities instead.

## Structural Modeling and Drug Bank

The RCSB protein data bank (PDB) is an open access resource in biology and medicine for finding three-dimensional structural data on large biological molecules such as proteins and can be used to find the PDB ID for the crystal structure of a protein of interest (Ex: HER2) [25]. All 3D structures found on this resource are experimentally verified by either X-ray crystallography or nuclear magnetic resonance (NMR) and give an accurate depiction of the structure of the protein and/or its binding domain. This makes it perfectly valid for *in silico* use and for extrapolating that data towards *in vitro* and *in vivo* studies. Furthermore, a comprehensive list of potential inhibitors, agonists, and antagonists can be obtained from a variety of existing sources. For example, the Natural Product Activity & Species Source Database (NPASS) can be used to find potential nutraceuticals that are effective against the protein of interest or use that data to develop a novel drug that is analogous in structure [26]. Alternatively, the DrugBank library, a comprehensive open access database containing information on drugs, drug properties, and drug targets, may be used to screen approved and experimental drugs to find effective inhibitors of the protein of interest [27].

**Figure 1:**
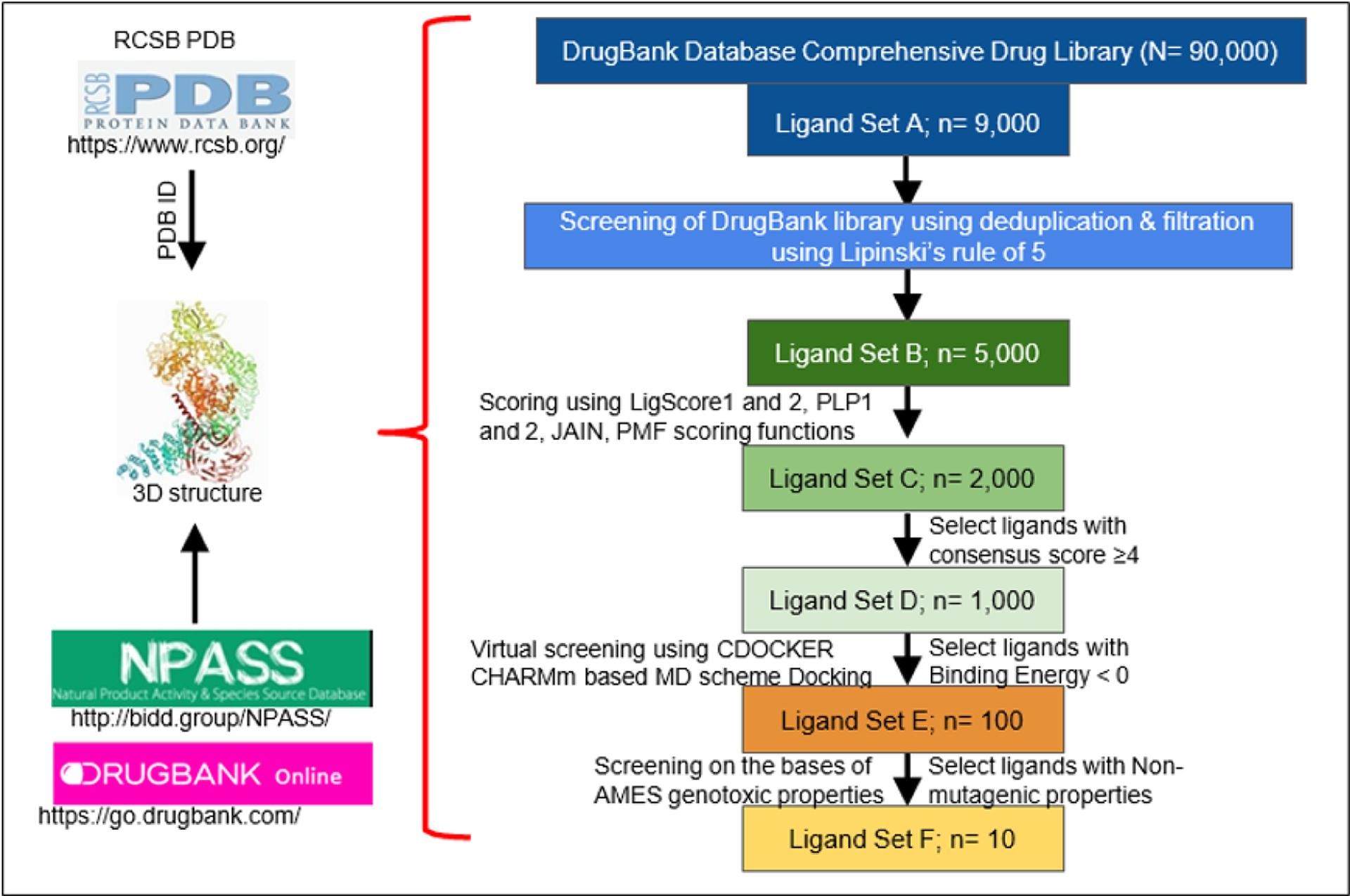
Steps in identification of drugs through structural modeling. RCSB PDB is analyzed for the specific protein ID. The 3D structure of the protein of interest, which is verified by X Ray crystallography or NMR, is used for further analysis. Identified binding domains are screened on different drug bank libraries on the basis of different parameters (n= is the depiction for the total target number).

This can all be accomplished in a matter of days or weeks by performing a multi-layered High-Throughput Virtual Screening (HTVS) with the BIOVIA Discovery Studio Client 2020 software. In a multi-layered HTVS, several screening layers are performed in succession to identify the best molecule that will bind to the protein of interest [28]. This process takes place in three stages, each of which sequentially narrows down the list of potential inhibitors. First, a preliminary rigid docking analysis, using the DS LibDock extension, takes place by comparing the binding energies of the protein’s crystalline structure to each ligand from the identified drug library in a rigid conformation to determine which ligands best fit at the binding site. Then, a flexible docking analysis, using the CDOCKER extension, takes place by mimicking the flexible nature of the binding site domain in nature and produces docked conformations with extreme precision. The number of potential drugs is then finalized after an Absorption, Distribution, Metabolism, Excretion, and Toxicity (ADMET) analysis is performed to determine the exact pharmacokinetic properties of the protein-drug interactions that were identified. Typically, after completion, anywhere from 10–20 drugs are identified and can then undergo further testing via *in vitro* and *in vivo* analyses to confirm their potential for inhibition. Figure 1 summarizes the steps in identification of drugs through structural modeling.

## Conclusion

Using these underutilized *in silico* tools will save a lot of time and money when considering the alternatives that are much more labor and resource intensive. For example, High-Throughput Screenings (HTS) are similar to HTVS except they are performed physically under wet-lab conditions. HTS is a drug discovery process that is popular amongst many pharmaceutical companies and takes advantage of robotics to autonomously screen a library of drugs and test their biological functions for pharmacological profiling [29]. The problem is that the equipment required (robots) and the bioactive drug screening libraries can cost tens of thousands of dollars, require technical training to use precisely, and can take months to finish a large screening analysis. Furthermore, from the ADMET analysis, HTVS can analyze pharmacokinetic parameters, like toxicity, of the identified drugs and their potential impact on certain tissues, like the liver, something which requires further testing after completion of HTS.

On top of all that, once this process is completed, a new drug is not discovered. Rather, these molecules are identified as “leads” for furthering and optimizing the drug discovery process, which takes too long to be feasible for immediate use. In fact, from lab experimentation to clinical testing and drug approval, novel drug development is a complex, time-consuming, and expensive process that can cost a manufacturer million, sometimes even billions [30] of dollars in resources and 12–15 years for completion [31]. By taking advantage of the latest bioinformatics techniques, such as HTVS, to analyze protein-drug interactions, small molecule inhibitors for cancer protein targets can be found with ease by repurposing existing drugs instead of waiting years for new drug approval. The potential for repurposing existing drugs as antagonists for novel cancer protein targets shows great promise and should be a more frequently explored option by pharmaceutical companies worldwide.

## Abbreviations:

HTVS: High-Throughput Virtual Screenings
AYA: Adolescence And Young Adult
ER: Estrogen Receptor
PR: Progesterone Receptor
HER2: Human Epidermal Growth Factor Receptor
MS: mass-Spectroscopy
PDB: Protein Data Bank
NMR: Nuclear Magnetic Resonance
NPASS: Natural Product Activity & Species Source Database
ADMET: Absorption, Distribution, Metabolism, Excretion, and Toxicity
